# Space–time interference between the length and duration of static lines

**DOI:** 10.3758/s13414-025-03093-8

**Published:** 2025-05-27

**Authors:** Daniel Bratzke, Rolf Ulrich

**Affiliations:** 1 Department of Psychology, University of Bremen, Bremen, Germany; 2 Department of Psychology, Eberhard Karls University of Tübingen, Tübingen, Germany

**Keywords:** Cross-dimensional interference, Time perception, Space–time interaction

## Abstract

The present study investigated space–time interference with static lines. In three preregistered online experiments, participants were presented with lines of different lengths and durations and asked to provide length or duration estimates after each trial on a visual analogue scale. Results showed bidirectional space–time interference in all experiments, with a slight asymmetry toward larger space-on-time than time-on-space interference when the target dimension was indicated only after stimulus presentation. Overall, the present findings add to a growing body of evidence demonstrating that space–time interference is more flexible than previously assumed.

How are space and time linked in our minds? Studies investigating space–time interference are a critical source of possible answers to this question. A prominent example is the study by Casasanto and Boroditsky (2008), who observed consistent evidence for asymmetric space–time interference in a series of six experiments, with space affecting time much more than vice versa. Specifically, they asked their participants to reproduce the length or the duration of lines (growing and static), which were presented with different lengths and for different durations. Their results showed that duration reproductions were affected by the irrelevant length, but length reproductions were not, or to a much lesser degree, influenced by the irrelevant duration. This asymmetric interference pattern has been interpreted within the conceptual metaphor theory framework (Lakoff & Johnson, 1980), which assumes that the mental representation of abstract concepts, such as time, is grounded in mental representations of other concrete dimensions, such as space. Accordingly, asymmetric space–time interference arises because when thinking or talking about time, people use the concrete dimension of space as a metaphor for the abstract dimension of time but not time to think about space (spatial metaphor account; see also Boroditsky, 2000). An alternative perspective on space–time interference is offered by “A Theory of Magnitude” (ATOM; e.g., Walsh, 2003), which posits that magnitudes such as space, number, and time share a common representational metric (Walsh, 2003). This fundamental assumption of ATOM is often regarded as consistent with symmetric rather than asymmetric space–time interference (e.g., Bottini & Casasanto, 2010; but see also Walsh, 2013).

Even though the asymmetric space–time interference pattern reported by Casasanto and Boroditsky (2008) has been replicated several times (Gijssels et al., 2013; Homma & Ashida, 2015; Merritt et al., 2010; Moon et al., 2015), an increasing number of recent studies challenges the assumption that space–time interference is always asymmetric and highlights the possibility of a more flexible relationship (Bratzke et al., 2024; Cai & Connell, 2015; Cai & Wang, 2022; Cai et al., 2018; Homma & Ashida, 2019; Vidaud-Laperrière et al., 2022; Whitaker et al., 2024; for a review, see Riemer & Cai, 2024). For example, Vidaud-Laperrière et al. (2022) even observed a reversed asymmetry—that is, stronger time-on-space than space-on-time interference—when they increased the difficulty of the spatial task (see also Homma & Ashida, 2019). In their study, participants had to judge the spatial or temporal distance between two successively presented dots. In this case, participants likely experience an apparent motion between the two dots (imputed motion; e.g., Jones & Huang, 1982), the speed of which is probably affected by the spatial distance as well as the temporal delay between the two dots. Consequently, spatial distance and speed of apparent (or imputed) motion are confounded in their study.

Such a confound naturally also exists with moving stimuli such as growing lines (e.g., Casasanto & Boroditsky, 2008; Whitaker et al., 2024) and may lead to duration judgments based on speed representations, thus a combination of duration and space information. For example, when reproducing the duration of a growing line, participants might mentally resimulate the growing line and terminate the duration reproduction when the simulated line has reached the same length as the remembered line (in the case of length reproduction, simply the final length of the line needs to be retrieved). If the representation of speed is subject to the same bias of central tendency (lower speeds are overestimated and higher speeds are underestimated) as duration (e.g., Bausenhart et al., 2014; Jazayeri & Shadlen, 2010; Vierordt, 1868), this would result in the usually observed space-on-time interference with longer duration estimates for longer lines. Specifically, consider two growing lines of different lengths presented for the same duration; the shorter line would grow at a lower rate than the longer one. If the shorter line is reproduced with a slightly higher speed and the longer line with a slightly lower speed (due to the central tendency bias), the reproduced duration would be longer for the longer line than for the shorter one, which corresponds to the usually observed space-on-time interference. Importantly, however, this would not indicate cross-dimensional interaction at a perceptual or representational level but rather reflect the use of speed information to make a duration judgment (for an alternative speed-related explanation of asymmetric space–time interference, see Riemer & Cai, 2024).

In the present study, we focused on static lines, which, unlike growing lines and moving dots, do not involve a confound with the speed of apparent or physical motion. In contrast to previous studies that used the reproduction or the bisection method, participants in the present study provided their spatial and temporal estimates on the same visual analogue scale (VAS; see, e.g., Bryce & Bratzke, 2015; Corallo et al., 2008) without absolute metric labels (the two ends were labeled “very short” and “very long”). We used this method to reduce the possibility that participants mentally resimulate the encoding situation during the reproduction. Such a mental simulation could be problematic. For example, the spatial information could be preserved in the simulation when reproducing time, whereas the time information could be lost when reproducing space because the spatial information is immediately available, and consequently there is no need to simulate it for the whole duration (see also Lambrechts et al., 2013). We consider the estimate provided via the VAS to be more abstract, similar to a verbal estimate (for direct comparisons between the VAS and reproduction methods, see Bryce & Bratzke, 2015; Damsma et al., 2021). Another advantage of the VAS is that both estimates are provided on the same (relative) scale, so that the two estimates can be better compared with each other.

Importantly, a recent preregistered replication of Casasanto and Boroditsky’s (2008) first experiment by Whitaker et al. (2024),^1^ did not provide consistent evidence for an asymmetric space–time interference pattern, even though it involved the original reproduction method. However, Whitaker et al. also used the same growing lines as in Casasanto and Boroditsky’s first experiment, which involved objective physical motion, and therefore the abovementioned confound between physical size and motion. Moreover, Whitaker et al. pointed out that different statistical analyses of space–time interference yielded different results in their study, with the best match between their and Casasanto and Boroditsky’s results, when they used ordinary least squares (OLS) regressions on grand average reproductions (averaged across participants). Therefore, we also used different statistical approaches in the present study to evaluate the robustness of the interference effects.

The present study comprised three experiments (see Fig. 1 for the time course of an experimental trial). In Experiment 1, the to-be-judged dimension (space or time) was only indicated after the presentation of the line (as in Experiment 1 of Casasanto & Boroditsky, 2008). In contrast, in Experiments 2 and 3, the relevant dimension was cued before each line presentation (as in Experiments 2 to 6 of Casasanto & Boroditsky). Experiment 3 differed from Experiments 1 and 2 in the range of tested durations, with the same range as in Casasanto and Boroditsky’s experiments in Experiment 3 (1,000–5,000 ms), and a smaller range in Experiments 1 and 2 (200–1,000 ms). If the mental representation of time is rooted in the mental representation of space, as the spatial metaphor account of space–time interference assumes, one would expect to replicate the asymmetric space–time interference observed by Casasanto and Boroditsky under all these conditions.

**Fig. 1:**
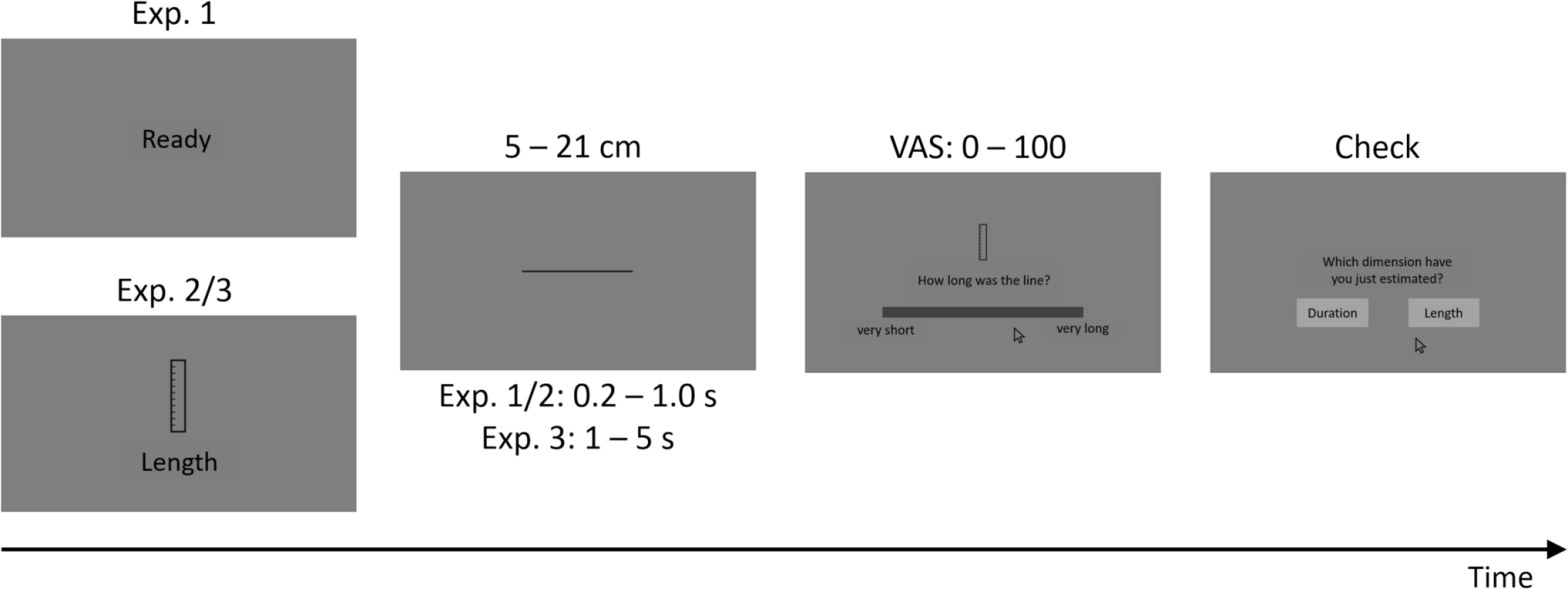
Schematic depiction of an exemplary experimental trial

## Experiment 1

In Experiment 1 (see https://aspredicted.org/2WD_YPG), static lines of different lengths and durations were presented, and participants estimated their length or duration on a VAS. To ensure that participants provided the estimate for the relevant dimension, they were asked after each trial to indicate which of the two dimensions they had estimated.

### Method

#### Participants

Twenty-five students of the University of Bremen participated for course credit. Based on the results of Casasanto and Boroditsky (2008), we expected a large effect size of at least *d* = 0.8. A power analysis with *d* = 0.8, alpha = .05, and power = .95 yielded a required sample size of *N* = 23. Two participants were excluded because they indicated they had estimated the irrelevant dimension in more than 20% of all trials. Thus, the final sample consisted of 23 participants with a mean age of 25.8 years (*SD* = 7.0; 19 women and four men). All participants provided informed consent before data collection.

#### Apparatus and stimuli

The experiment was conducted online and ran on the participants’ computers. It was created in PsychoPy (Peirce et al., 2019) and hosted by Pavlovia (https://pavlovia.org). The PsychoPy code *ScreenScale* (Morys-Carter, 2021) was used to adjust the screen scale on individual computers. Test stimuli were nine horizontal lines with a length between 5 and 21 cm (with 2 cm between lengths), which were presented for one of nine durations between 200 and 1,000 ms (with 100 ms between durations). Length and duration judgments were assessed on a horizontal VAS (20 cm, ranging from 0 to 100) labeled “very short” at the left end and “very long” at the right end.

#### Tasks and procedure

Each trial started with a presentation of the word “Ready” at the center of the screen for 2 s. After another 250 ms, the line appeared at the center of the screen, and 500 ms after that, the VAS was presented in the lower part of the screen, together with the symbolic depiction (above the VAS) of a ruler and the question “How long was the line?” or an hourglass and the question “How long was the line presented for?” When the participant clicked on the VAS, a small red vertical line briefly indicated the marked position. After a delay of 250 ms with a blank screen, the question “Which dimension have you just estimated?” appeared, and participants had to provide their answer by clicking on one of two buttons (“duration” vs. “length”) below the question. Each combination of the nine line lengths and the nine durations was tested twice, once for each type of task (length vs. duration discrimination). Thus, there were 162 experimental trials in total.

### Results and discussion

Trials in which participants indicated that they had estimated the wrong dimension were excluded from all analyses (2.2% of all trials). We then calculated individual linear regressions for mean duration and mean line length estimates separately for the two predictors, duration and line length. For the cross-dimensional comparison of regression slopes, the predictor values were scaled so that their range corresponded to the range of the VAS (0–100),^2^ and *t* tests were used to compare regression slopes and *R*^2^s between the two cross-dimensional conditions. In addition to this preregistered analysis, we conducted three supplemental analyses: a multiple regression on mean duration and length estimates as in Cai and Wang (2022), linear mixed-effect (LME) models on the nonaggregated estimates from individual trials as in Whitaker et al. (2024), and OLS regressions on grand average estimates (averaged across individual trials and participants). The LME analysis was conducted using the “lmer” function from the R package *lme4* (Bates et al., 2015) and semipartial marginal *R*^2^s were computed with the R package *partR2* according to Stoffel et al. (2021). Separate LME analyses were conducted for duration and length estimates, with scaled duration and line length as fixed effects and participant as random effect. For the supplemental analyses, we only report the cross-dimensional interference effects. To assess the difficulty of each task, we calculated the mean coefficient of variation (CV) for duration and length estimates. The individual mean CVs of each estimate were calculated by dividing the standard deviation by the mean for each relevant stimulus condition and then averaging across conditions.

**Fig. 2:**
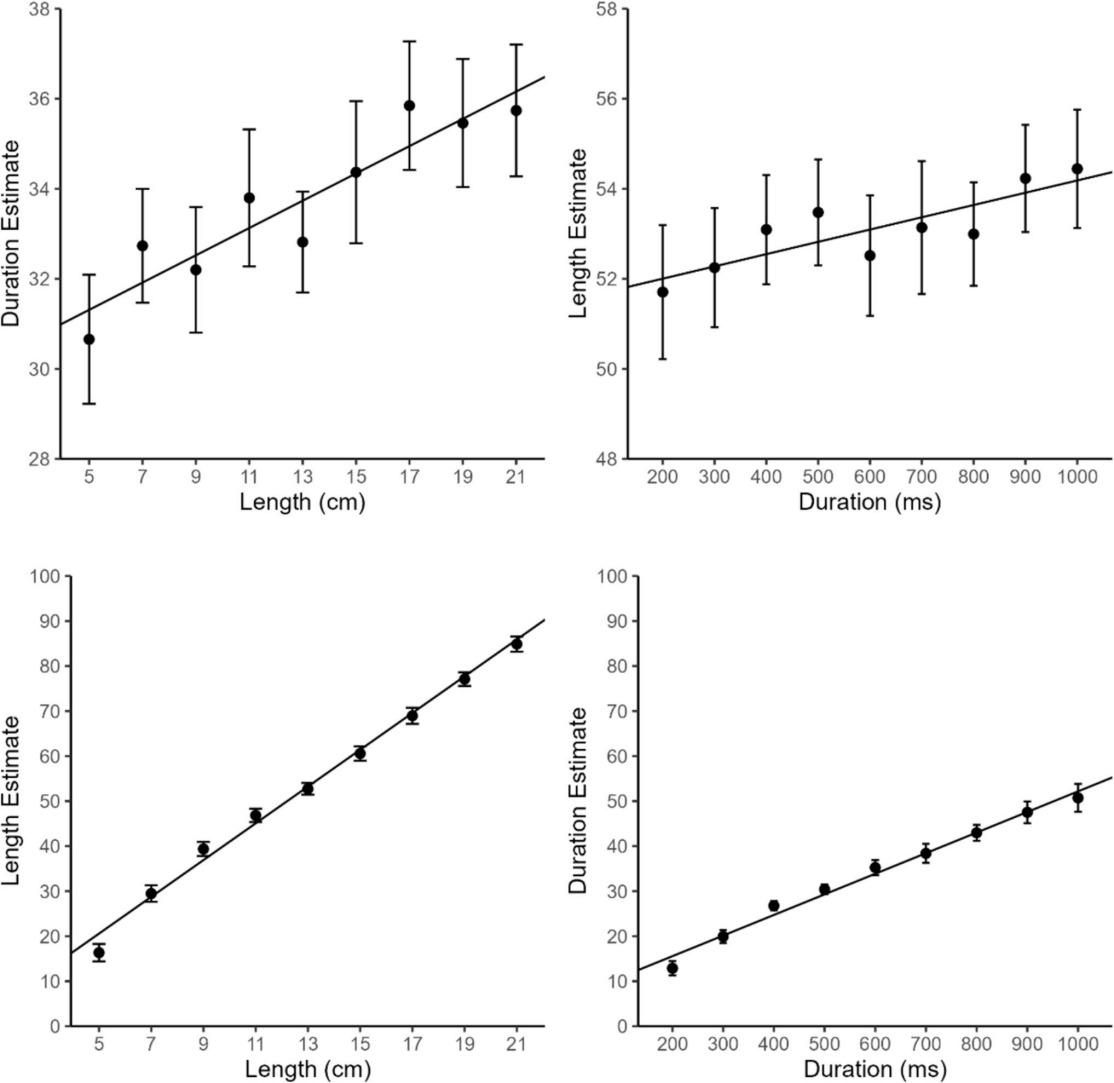
Mean duration and length estimates as a function of duration and line length in Experiment 1. Error bars represent ±1 within-subject *SE* according to Morey (2008). Regression lines show overall regressions with grand-averaged estimates as outcome variable. Duration and length estimates could range from 0 (“very short”) to 100 (“very long”)

As seen in the lower part of Fig. 2, participants apparently understood the task and adhered to the instructions. Nevertheless, the within-dimensional mean slope was larger for length than for duration estimates (0.653 vs. 0.366), *t*(22) = 7.09, *p* < .001, *d*_z_ = 1.48. This is not so surprising as there was a spatial frame of reference with an upper limit for line length imposed by the monitor but no such frame for duration. Within-dimensional mean *R*^2^s were high for both dimensions (length: .953 vs. duration: .893), but nevertheless differed significantly from each other, *t*(22) = 2.70, *p* < .013, *d*_z_ = 0.56. There was also a positive relationship between the two stimulus dimensions in both cross-dimensional cases (see upper part of Fig. 2). The mean regression slope was about two times larger for the space-on-time (0.048) than for the time-on-space (0.022) interference, *t*(22) = 2.08, *p* = .050, *d*_z_ = 0.43. Nevertheless, both slopes significantly differed from zero (space-on-time: *p* < .001; time-on-space: *p* = .008). Mean *R*^2^ was slightly larger for the space-on-time (.196) than for the time-on-space (.140) interference, even though not significantly different, *t*(22) = 1.03, *p* = .317, and both *R*^2^s differed significantly from zero (space-on-time: *p* < .001; time-on-space: *p* = .002).

The results of the multiple regression analysis essentially confirmed these results. In both cross-dimensional conditions, the irrelevant dimension turned out to be a significant predictor, but space-on-time interference was stronger than time-on-space interference (space-on-time: ß = 0.049, partial *R*^2^ = .204, *p* < .001; time-on-space: ß = 0.019, partial *R*^2^ = .053, *p* = .041). The LME analysis also confirmed the results of our main analysis, with ß = 0.075, 95% CI [0.041, 0.104] and semipartial *R*^2^ = .006 [95%CI: .000, 0.045] for space-on-time interference, and ß = 0.025, 95% CI [0.006, 0.047] and semipartial *R*^2^ < .001, 95% CI [.000, .022] for time-on-space interference. OLS regressions on grand average estimates showed similar effects (space-on-time: ß = 0.049, *R*^2^ = .863, *p* < .001; time-on-space: ß = 0.022, *R*^2^ = .715, *p* = .004), even though the difference between *R*^2^s for the two cross-dimensional conditions was less pronounced, similar to the results of our main analysis. The mean CV was larger for the duration (0.392) than for the length estimates (0.227), *t*(22) = 9.05, *p* < .001, *d*_z_ = 1.89, suggesting that the duration task was more difficult than the length task.

In sum, Experiment 1 showed larger space-on-time than time-on-space interference, consistent with Casasanto and Boroditsky’s (2008) observation of asymmetric space–time interference. However, the asymmetry in the present interference was rather subtle in comparison to their experiments, as we observed in most analyses rather similar *R*^2^s for the two cross-dimensional interferences, while they observed *R*^2^s that were, on average, 12 times larger for the space-on-time than for the time-on-space interference (ranging from 3 to 19 times larger; see their Fig. 3).

## Experiment 2

In Experiment 1, participants did not know which of the two dimensions would be task relevant until after the line presentation. Any cross-dimensional interference observed in this situation might be caused by the requirement to encode the information from both dimensions. Casasanto and Boroditsky (2008) observed the most substantial asymmetry in their Experiment 1, where participants were also not informed about the task-relevant dimension before the trial. If the relevant dimension to be judged is unknown, attention must be divided between the two dimensions. It is conceivable that the optimal allocation of limited attentional resources leads to conflict in processing the two dimensions, which might cause this asymmetric effect. In Experiment 2 (see https://aspredicted.org/M3K_NQ6), we tried to rule out this possible conflict by announcing the relevant dimension to be judged before presenting the line.

### Method

#### Participants

As in Experiment 1, 25 students of the University of Bremen participated for course credit. None of the participants was excluded from analyses due to an excessive error rate (>20%). The mean age was 23.8 years (*SD* = 4.4; 21 women, three men, and one nonbinary). All participants provided informed consent before data collection.

#### Apparatus and stimuli

The stimuli were the same as in Experiment 1. Again, the experiment was run on the participants’ individual computers.

#### Tasks and procedure

The tasks and procedure were identical to Experiment 1, with the following exception: At the beginning of each trial, participants were informed about the relevant task dimension. Accordingly, each trial started with a presentation of either the word “Length” together with the symbolic depiction of a ruler or the word “Duration” together with the symbolic depiction of an hourglass (instead of the word “Ready”) at the center of the screen for 2 s.

### Results and discussion

**Fig. 3:**
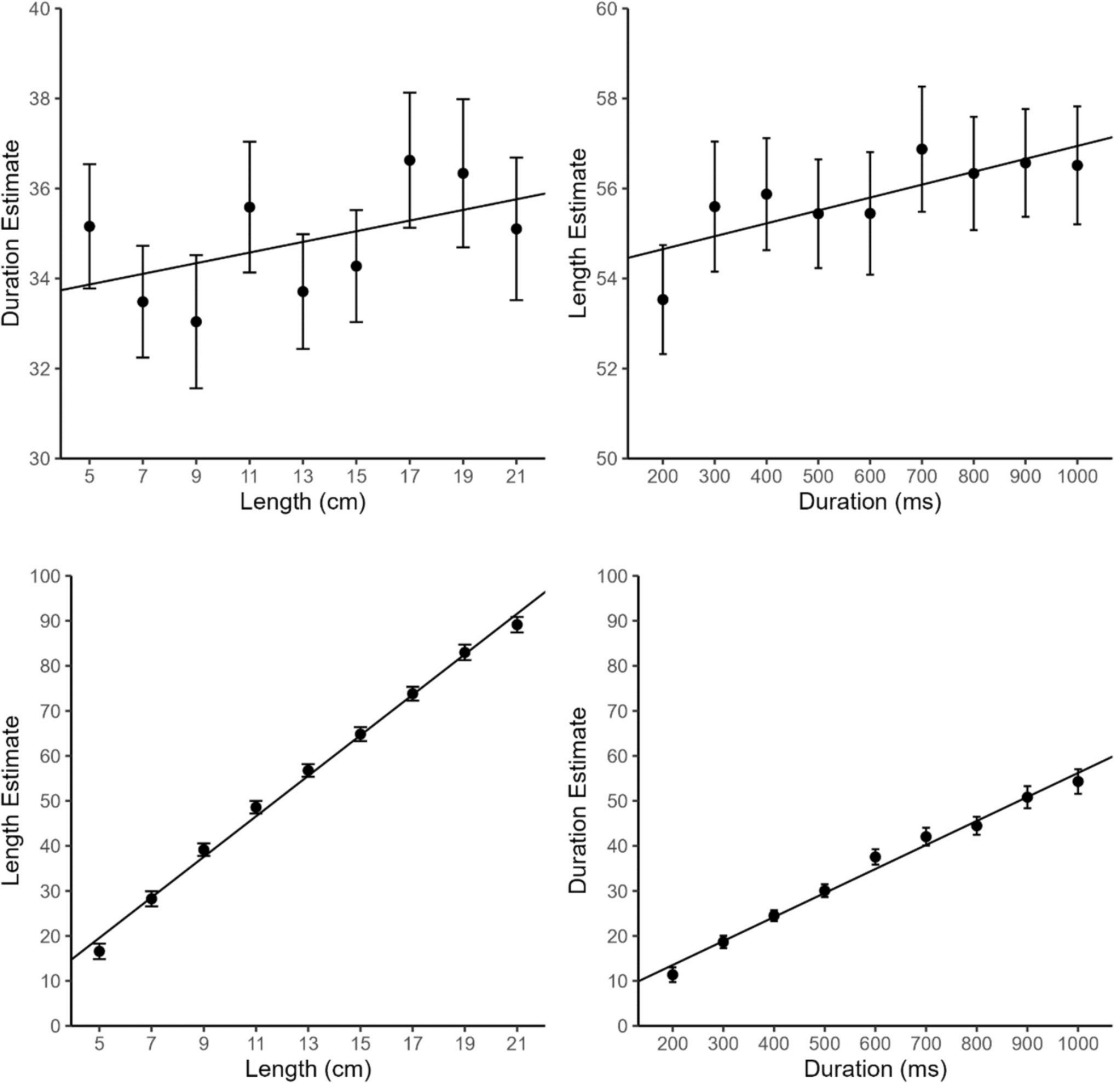
Mean duration and length estimates as a function of duration and line length in Experiment 2. Error bars represent ±1 within-subject *SE* according to Morey (2008). Regression lines show overall regressions with grand-averaged estimates as outcome variable. Duration and length estimates could range from 0 (“very short”) to 100 (“very long”)

Again, trials in which participants indicated that they had estimated the wrong dimension were excluded from all analyses (2.1% of all trials). As shown in the lower part of Fig. 3, the within-dimensional effects were very similar to those in Experiment 1. The mean slope was again larger for length than for duration estimates (0.720 vs. 0.427), *t*(24) = 8.26, *p* < .001, *d*_z_ = 1.65, and the same difference was again also observed for mean *R*^2^ (length: .967, duration: 0.896), *t*(24) = 4.93, *p* < .001, *d*_z_ = 0.99. As in Experiment 1, there was also a positive relationship between the two stimulus dimensions in the cross-dimensional cases (see upper part of Fig. 3). In contrast to Experiment 1, however, the mean regression slope was similar for the space-on-time (0.019) and for the time-on-space (0.023) interference, *t*(24) = 0.25, *p* = .802, *d*_*z*_ =0.15. Only the slopes for the time-on-space interference differed significantly from zero (*p* = .004; space-on-time: *p* = .127). Also, in contrast to Experiment 1, there was no significant difference between mean *R*^2^ for the two cross-dimensional conditions, *t*(24) = 0.74, *p* = .464, *d*_*z*_ = 0.05. Mean *R*^2^ was descriptively even slightly smaller for the space-on-time (.148) than for the time-on-space (.192) interference. However, as in Experiment 1, both *R*^2^s differed significantly from zero (*p* values < .001).

The results of the multiple regression analysis largely confirmed these results. In both cross-dimensional cases, the irrelevant dimension turned out to be a significant predictor, and space-on-time interference was slightly weaker than time-on-space interference (space-on-time: ß = 0.020, partial *R*^2^ = .051, *p* = .045; time-on-space: ß = 0.021, partial *R*^2^ = .081, *p* = .011). The LME analysis revealed considerably weaker space-on-time than time-on-space interference, with ß = 0.028, 95% CI [0.001, 0.054] and semipartial *R*^2^ = .289, 95% CI [.253, 0.323] for space-on-time interference, and ß = 0.026, 95% CI [0.011, 0.043] and semipartial *R*^2^ = .581, 95% CI [.545, .603] for time-on-space interference. OLS regressions on grand average estimates also revealed weaker space-on-time than time-on-space interference (space-on-time: ß = 0.019, *R*^2^ = .262, *p* = .159; time-on-space: ß = 0.023, *R*^2^ = .617, *p* = .012). The mean CV was again larger for the duration (0.392) than for the length estimates (0.188), *t*(24) = 7.47, *p* < .001, *d*_z_ = 1.49.

In contrast to Experiment 1, Experiment 2 exhibited a more symmetric cross-dimensional interference pattern, with both dimensions affecting each other. If anything, then the results show a reversed asymmetry with larger time-on-space than space-on-time interference as evidenced by the LME and grand average regression analyses. In comparison with Experiment 1, it seems that prior knowledge about, and therefore likely also attention to, the relevant task dimension attenuated particularly the space-on-time interference. This is inconsistent with Casasanto and Boroditsky’s (2008) results, where prior knowledge about the task-relevant dimension neither balanced the asymmetry nor significantly reduced the space-on-time interference, although the asymmetry between space-on-time and time-on-space interference was slightly reduced (see the results of Experiment 1 and Experiment 2 in their Fig. 3).

## Experiment 3

In Experiment 2, we observed relatively symmetric space–time interference, contrasting with the previous observations of asymmetric space–time interference by Casasanto and Boroditsky (2008). In Experiment 3 (https://aspredicted.org/T7L_G9L), we explored whether the range of tested durations, and therefore probably also the saliency (see Homma & Ashida, 2019) and the cognitive demands of the duration estimation task (see Vidaud-Laperrière et al., 2022), might be a crucial factor for the difference between the present and Casasanto and Boroditsky’s results. For example, Vidaud-Laperrière et al. (2022) observed larger time-on-space interference when they decreased the range of spatial distances. Similarly, Homma and Ashida (2019) observed larger time-on-space than space-on-time interference with a very small range of line lengths. They suggested that the relative saliency of spatial and temporal information affects the balance of space–time interference. The less difficult a task, and therefore more salient the task-related information, the stronger it should interfere with the other dimension.

The range of durations used in the first two experiments of the present study (200–1,000 ms) was considerably smaller and, on average, shorter than that of Casasanto and Boroditsky’s (2008) study (1,000–5,000 ms). Consequently, the difficulty of the duration task could differ between these two ranges and therefore affect the balance of space–time interference. More specifically, a larger range of durations with, on average, longer durations could decrease the saliency of the temporal information (and increase the difficulty of the duration task) and therefore reduce time-on-space interference. In Experiment 3, we replicated Experiment 2 with exactly the same durations as in Casasanto and Boroditsky’s study.

### Method

#### Participants

As in the previous experiments, 25 students of the University of Bremen participated for course credit. None of the participants had to be excluded from analyses due to an excessive error rate (>20%). The mean age was 24.8 years (*SD* = 5.1; 19 women, six men). All participants provided informed consent before data collection.

#### Apparatus and stimuli

The stimuli were the same as in Experiment 1, except that the nine tested durations ranged from 1,000 to 5,000 ms (with 500 ms between durations). Again, the experiment was run on the participants’ individual computers.

#### Tasks and procedure

Tasks and procedure were identical to those of Experiment 2.

### Results and discussion

**Fig. 4:**
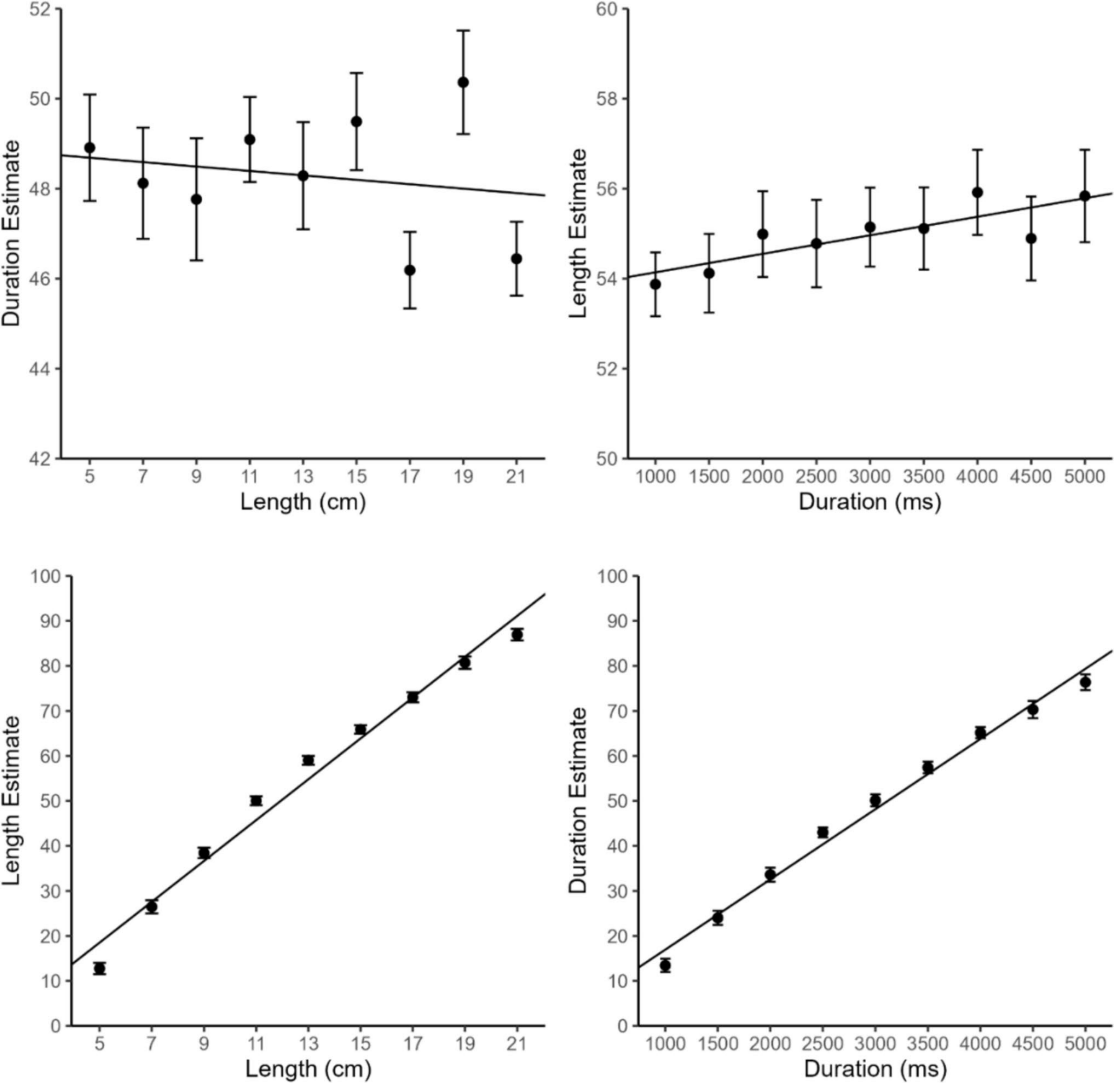
Duration and length estimates as a function of duration and line length in Experiment 3. Error bars represent ±1 within-subject *SE* according to Morey (2008). Regression lines show overall regressions with grand-averaged estimates as outcome variable. Duration and length estimates could range from 0 (“very short”) to 100 (“very long”)

We excluded 2.15% of all trials from all analyses because participants indicated they had estimated the wrong dimension. The within-dimensional relationships were more similar between the two dimensions than in Experiments 1 and 2 (see lower part of Fig. 4). The mean slope was nevertheless still significantly larger for length (0.726) than for duration (0.624) estimates, *t*(24) = 4.11, *p* < .001, *d*_*z*_ = 0.82, and mean *R*^2^ also differed between the two dimensions (length: .960 vs. duration: .941),* t*(24) = 2.54, *p* = .018, *d*_*z*_ = 0.51. Considering the cross-dimensional interference (see upper part of Fig. 4), there was again a positive relationship for the time-on-space interference (slope: 0.016). However, the space-on-time interference showed a rather inconsistent pattern,^3^ with an even negative slope (−0.008), which differed significantly from the time-on-space slope, *t*(24) = 2.28, *p* = .032, *d*_*z*_ = 0.46. As in Experiment 2, only the time-on-space slope differed from zero (*p* = .027; space-on-time: *p* = .425). Also consistent with Experiment 2, there was no significant difference between mean *R*^2^ for the two cross-dimensional conditions, *t*(24) = 1.44, *p* = .162, *d*_*z*_ = 0.29. Again, mean *R*^2^ was descriptively even slightly smaller for the space-on-time (.109) than for the time-on-space (.166) interference. As in all previous experiments, both *R*^2^s differed significantly from zero (*p* values < .001).

In the multiple regression analysis, neither duration nor line length turned out to be a significant predictor for line and duration estimates (space-on-time: ß = −0.013, partial *R*^2^ = .017, *p* = .253; time-on-space: ß = 0.013, partial *R*^2^ = .014, *p* = .297). In contrast to these results, the LME analysis revealed somewhat weaker space-on-time than time-on-space interference, with ß = −0.14, 95% CI [−0.044, 0.010] and semipartial *R*^2^ = .483, 95% CI [.460, .515] for space-on-time interference, and ß = 0.017, 95% CI [0.003, 0.038] and semipartial *R*^2^ =.613, 95% CI [.599, .631] for time-on-space interference. OLS regressions on grand-average estimates showed an even stronger asymmetry, with significant interference only for time on space (space-on-time: ß = −0.008, *R*^2^ = .039, *p* = .611; time-on-space: ß = 0.017, *R*^2^ = .694, *p* = .005). The mean CV was slightly smaller than in the previous experiments for the duration estimates (0.311 vs. 0.392 in Experiments 1 and 2) but very similar for the length estimates (0.208 vs. 0.227 in Experiment 1 and 0.188 in Experiment 2). Nevertheless, the mean CV still differed between the two dimensions, *t*(24) = 5.43, *p* < .001, *d*_z_ = 1.09.

The extended duration range in Experiment 3 resulted in an inconsistent space-on-time pattern with only very little evidence for space-on-time interference. Notably, the time-on-space interference was not affected similarly by the extension of the duration range, again showing a small but reliable positive interference effect (only the multiple regression analysis revealed no significant time-on-space interference), although the difficulty of the duration task (as indexed by the CV) was even slightly lower than in the previous experiments. Overall, the results of Experiment 3 suggest that the discrepancy between the results of Casasanto and Boroditsky (2008) and the present Experiment 2 is not due to the differences in the employed duration range.

## General discussion

The present study investigated space–time interference between the length and duration of static lines. The results showed little evidence for an asymmetric interference pattern (larger space-on-time than time-on-space interference), as previously reported by Casasanto and Boroditsky (2008). In Experiment 1, in which participants had no prior knowledge about the relevant, to-be-estimated dimension, the regression slope indeed indicated an asymmetry, with two times larger slopes for the space-on-time than for the time-on-space interference (see also Merritt et al., 2010). Although no significant difference in the strength of the relationship (i.e., the coefficient of determination, *R*^2^) was observed in our main analysis for Experiment 1, supplemental analyses consistently showed larger *R*^2^s for space-on-time than for time-on-space interference. When participants were informed about the relevant dimension before each estimation in Experiments 2 and 3, interference was either relatively symmetric (Experiment 2) or slightly reversed (Experiment 3). Thus, space–time interference seems not to be obligatorily asymmetric but relatively flexible (see also Cai & Connell, 2015; Cai & Wang, 2022; Homma & Ashida, 2019; Vidaud-Laperrière et al., 2022). Table 1 presents a comprehensive summary of the evidence derived from our primary analyses based on individual regressions.

**Table 1:** Evidence from primary analyses (individual regressions)

Experiment		Interference	
	Space-on-Time (ST)	Time-on-Space (TS)	Asymmetry (ST >TS)
Experiment 1			
Slope	Yes	Yes	Yes
*R*^2^	Yes	Yes	No
Experiment 2			
Slope	No	Yes	No
*R*^2^	Yes	Yes	No
Experiment 3			
Slope	No	Yes	No
*R*^2^	Yes (Negative)	Yes	No

### Possible factors that influence the (a)symmetry of space–time interference

In the present study, we used static lines to eliminate a potential confound between duration and motion speed, which could affect duration estimates with growing lines (e.g., Casasanto & Boroditsky, 2008; Vidaud-Laperrière et al., 2022). We reasoned that when estimating the duration of a growing line, participants might combine speed and length information instead of relying solely on a duration representation. The exclusion of speed as a potential source of interference could thus explain why we did not observe the strong asymmetric interference pattern of Casasanto and Boroditsky (2008). However, others have reported asymmetric space–time interference even with static lines (Casasanto & Boroditsky, 2008; Homma & Ashida, 2015; Merritt et al., 2010). Whether speed information plays a significant role in (asymmetric) space–time interference, and if so, how it influences temporal and spatial representations (and judgments) remains an open question (see also Riemer & Cai, 2024).

Recently, several ideas have been proposed on how the (a)symmetry of space–time interference might be modulated (see Riemer & Cai, 2024). Cai et al. (2018) proposed a Bayesian inference account of space–time interference (see also Lambrechts et al., 2013), which assumes that when providing a judgment about the magnitude of a particular dimension, the representations of space and time information are recalled and integrated, based on the prior belief that the two dimensions are positively correlated (e.g., a longer distance takes longer to travel). This model predicts that the susceptibility of the target dimension to interference from the irrelevant dimension increases with its representational noise (see also Cai & Wang, 2022). Consistent with this prediction, Homma and Ashida (2019) suggested that the saliency of signals might be crucial for the balance of cross-dimensional interactions, with higher saliency (and therefore probably also lower representational noise) resulting in stronger interference. In all three experiments of the present study, task difficulty (as indexed by the CV) was higher for the temporal than for the spatial task, which, according to the ideas of Cai et al. and Homma and Ashida, should have led to larger space-on-time than time-on-space interference (as in Casasanto & Boroditsky, 2008). Overall, it seems that task difficulty (or the saliency of information) did not play a major role in the present study. However, all considerations regarding the role of task difficulty in the present study are certainly limited as there was generally little variation in task difficulty.

Some researchers have suggested that the asymmetry of space–time interference results from differences in the automaticity of processing between the two dimensions, assuming that spatial information is processed automatically, whereas temporal information is not (Dormal & Pesenti, 2013; Riemer et al., 2024; Starr & Brannon, 2016). The present results are rather ambiguous concerning this automaticity account. On the one hand, it is supported by the result that when space and time compete for attentional resources (as in Experiment 1), space processing seems to dominate (i.e., larger space-on-time than time-on-space interference). On the other hand, when attention can be directed to the relevant dimension (space or time; as in Experiments 2 and 3), irrelevant time seems to be processed to a similar degree as irrelevant space (i.e., similar space-on-time and time-on-space interference). Thus, the space–time asymmetry seems to decrease when the demand to focus on the two dimensions simultaneously no longer exists, which is rather inconsistent with the automaticity account.

### What causes time-on-space interference?

Vidaud-Laperrière et al. (2022) suggested that for time-on-space interference to occur, the spatial information must be of rather low saliency (hindering automatic spatial processing) and additionally of sequential nature (e.g., growing lines or distances marked by sequential dots). The present results with static lines clearly demonstrate that a sequential nature of the spatial information (e.g., growing lines) is not a prerequisite for time-on-space interference. Of course, this does not exclude the possibility of even stronger time-on-space interference with moving stimuli, for example, because presenting spatial information sequentially may balance the working memory demands across the two dimensions, which are presumably much lower in the spatial domain when the spatial information is immediately accessible (Vidaud-Laperrière et al., 2022; see also Cai et al., 2018). Time-on-space interference with static lines was previously also reported by Homma and Ashida (2019). These authors observed time-on-space interference in the absence of space-on-time interference (similar to our results of Experiment 3) when they used a challenging line estimation task (with a very small length range of 12 pixels). While these results can be viewed as evidence that space–time interference can be flexible across the whole range of asymmetries also with static spatial information, it should be treated with caution. In Homma and Ashida’s study, the spatial task was extremely difficult (performance was close to chance level), and therefore participants probably had to, at least partly, rely on the irrelevant duration information to provide a spatial judgment (see also Vidaud-Laperrière et al., 2022). Although this was certainly not the case in Experiment 3 of the present study, the space-on-time interference in that experiment showed a somewhat inconsistent, nonlinear relationship, which is difficult to interpret.

## Limitations

The present study introduced a new method to investigate cross-dimensional space–time interference, the VAS. We reasoned that this method would minimize a possible confound associated with the reproduction method—namely, that participants mentally resimulate the encoding situation during their reproduction. More specifically, such mental resimulation likely incorporates the length information when reproducing duration, while the duration information might get lost during the length reproduction. The use of the VAS, however, might have introduced a new confound—namely, that both estimates were provided on the same spatial scale. In order to provide a duration estimate on this scale, an internal duration representation needs to be transformed into a spatial distance, which we assumed participants would be able to do fairly intuitively. However, it cannot be ruled out that during that transformation spatial information is taken into account, because the spatial nature of the scale somehow suggests this. If this was the case, however, we should have observed consistent space-on-time interference in all experiments, which was not the case. One could also suspect that participants in the length estimation task directly mapped the length of the presented line onto the VAS scale (similar to the length reproduction in Casasanto & Boroditsky, 2008). In this case, the longest line (21 cm) should be estimated near the higher end of the scale, whereas the shortest line (5 cm) should never be estimated near the lower end. This pattern was not observed, as there was a rather symmetric central tendency effect for line length estimates, regardless of the scale’s ends. Moreover, we do not see any plausible reason why the spatial nature of the scale should have induced time-on-space interference, as in this case, spatial information needs to be transformed into a relative spatial estimate. Therefore, while we cannot rule out that the use of the VAS affected the result pattern in some way, we believe that it did not result in a systematic confound.

### The role of different statistical approaches

As already mentioned in the Introduction, Whitaker et al. (2024) pointed out that different statistical approaches have been used to investigate space–time interference, which led to different results in their recent direct replication of Casasanto and Boroditsky’s (2008) Experiment 1. While predictor estimates (betas) were relatively invariant to the method, some of the *R*^2^ values changed dramatically depending on preanalysis data averaging (no averaging, person-level, or grand average). The present study partly showed similar discrepancies between the different methods, most pronounced in the results of Experiment 3, where the LME analysis on nonaveraged data showed much higher *R*^2^s than all other analyses, and the difference in *R*^2^s between the two kinds of interference from the OLS regressions was considerably larger than for all other methods. Importantly, in the present study the different analyses did not lead to fundamentally different conclusions overall.

## Conclusion

The present study did not show the strong asymmetric space–time interference pattern reported by Casasanto and Boroditsky (2008) with nonlinguistic stimuli. The present results add to a growing body of evidence demonstrating that space–time interference is not always asymmetric but rather flexible (see Riemer & Cai, 2024). Taken together with previous findings of bidirectional, or even reversed asymmetric interference patterns, the present results suggest that space–time interference with nonlinguistic stimuli may be distinct from similar interference with linguistic or other symbolic stimuli (e.g., Birngruber & Ulrich, 2019; Bottini & Casasanto, 2010; Janczyk et al., 2023). While the latter phenomena may be explained by conceptual metaphor theory, the former may be related to interference in a perceptual memory (see also Winter et al., 2015), perhaps facilitated by a common representational metric for magnitude (Walsh, 2003).

## Data Availability

The datasets generated during and/or analyzed during the current study are available via OSF (https://osf.io/wcv6f/)*.*

## Footnotes

1 The results of Whitaker et al.’s (2024) study were not yet published when we planned and conducted the present study.

2 This scaling method yields the same results as when the scores were *z* transformed, except for a linear transformation of the regression slopes.

3 To further explore the inconsistency in the space-on-time interference pattern, we inspected the individual regressions. Sixteen (9) participants showed a negative (positive) regression slope, and only two participants showed the same up-and-down pattern visible in the mean pattern for the length range from 7 to 21 cm.
